# Twenty Years of Blast-Induced Neurotrauma: Current State of Knowledge

**DOI:** 10.1089/neur.2024.0001

**Published:** 2024-03-14

**Authors:** Tarun Sachdeva, Shailesh G. Ganpule

**Affiliations:** ^1^ Department of Mechanical and Industrial Engineering, Indian Institute of Technology Roorkee, Roorkee, India.; ^2^ Department of Design, Indian Institute of Technology Roorkee, Roorkee, India.

**Keywords:** animal models, behavioral outcomes, biomechanics, clinical studies, pathology, primary blast

## Abstract

Blast-induced neurotrauma (BINT) is an important injury paradigm of neurotrauma research. This short communication summarizes the current knowledge of BINT. We divide the BINT research into several broad categories—blast wave generation in laboratory, biomechanics, pathology, behavioral outcomes, repetitive blast in animal models, and clinical and neuroimaging investigations in humans. Publications from 2000 to 2023 in each subdomain were considered. The analysis of the literature has brought out salient aspects. Primary blast waves can be simulated reasonably in a laboratory using carefully designed shock tubes. Various biomechanics-based theories of BINT have been proposed; each of these theories may contribute to BINT by generating a unique biomechanical signature. The injury thresholds for BINT are in the nascent stages. Thresholds for rodents are reasonably established, but such thresholds (guided by primary blast data) are unavailable in humans. Single blast exposure animal studies suggest dose-dependent neuronal pathologies predominantly initiated by blood–brain barrier permeability and oxidative stress. The pathologies were typically reversible, with dose-dependent recovery times. Behavioral changes in animals include anxiety, auditory and recognition memory deficits, and fear conditioning. The repetitive blast exposure manifests similar pathologies in animals, however, at lower blast overpressures. White matter irregularities and cortical volume and thickness alterations have been observed in neuroimaging investigations of military personnel exposed to blast. Behavioral changes in human cohorts include sleep disorders, poor motor skills, cognitive dysfunction, depression, and anxiety. Overall, this article provides a concise synopsis of current understanding, consensus, controversies, and potential future directions.

## Introduction

**FIG. 1.**
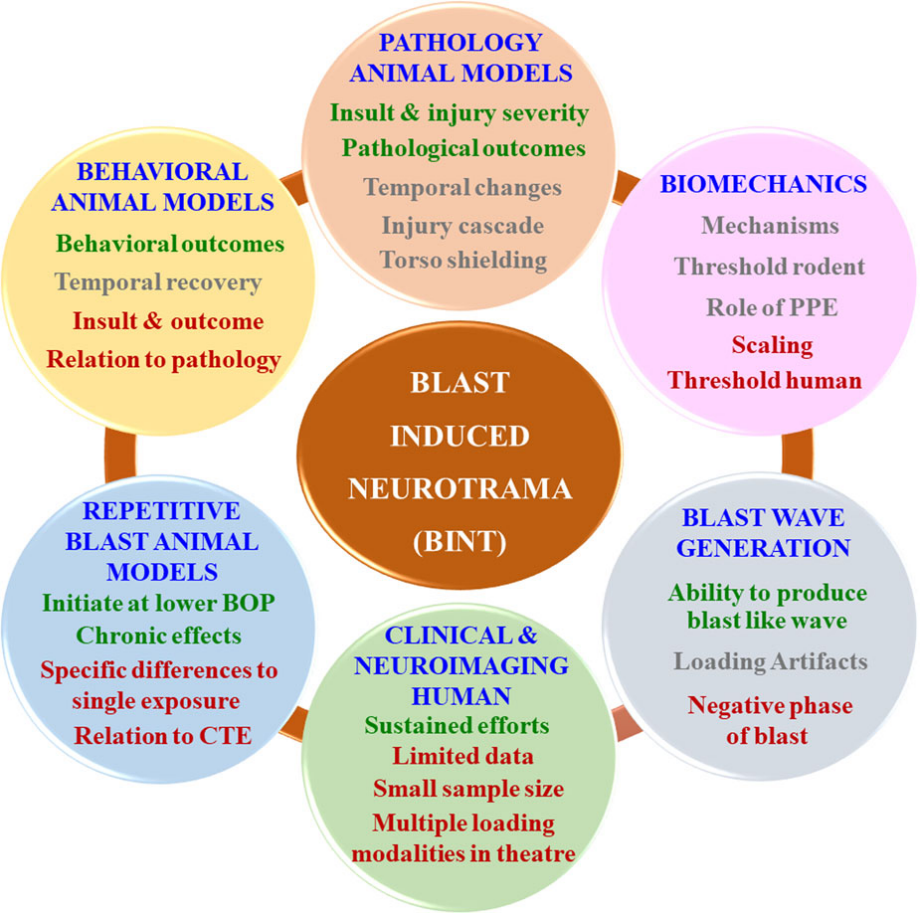
Schematic depicting the current state of knowledge of BINT in various subdomains. The text color for each aspect in individual subdomains is commensurate with the degree of understanding. Green-colored text indicates significant, unequivocal findings in the literature. Gray-colored text indicates that the findings are equivocal. Red-colored text indicates a lack of substantial literature. For example, in the “behavioral animal models” subdomain, unequivocal findings are reported in the literature for various behavioral outcomes observed because of exposure to the blast demonstrating/exuberating consensus across investigations. However, findings regarding temporal recovery of the behavioral outcomes are equivocal (or confounding), indicating a lack of consensus. There is a lack of substantial literature regarding the relationship between external mechanical insult and behavioral outcomes and the correlation between observed pathology and behavioral outcomes. Future work in each subdomain should focus on aspects highlighted using gray- and red-colored texts. BINT, blast-induced neurotrauma; BOP, blast overpressure; CTE, chronic traumatic encephalopathy; PPE, personal protective equipment.

Blast-induced neurotrauma (BINT) has been identified as a signature wound during conflicts in Iraq and Afghanistan.^1,2^ More than 20 years have passed since the early research publications of BINT.^3–5^ Since then, significant efforts have been made to understand BINT. In the past 20 years, ∼$900 million USD has been spent on research funding related to BINT.^6^ In this short communication, we summarize (Fig. 1) the current knowledge, consensus, controversies, and potential future directions. Additional details regarding this short communication are provided in the section Transparency, Rigor, and Reproducibility Summary at the end of the article.

## Blast Wave and Its Generation in a Laboratory

An explosion is termed as a rapid expansion of gases, generally occurring because of the detonation of explosives.^7^ Explosion creates a pressure pulse in the surrounding medium (e.g., air) that propagates at supersonic speed, generating an (almost) instantaneous rise in pressure known as a shock front. The rarefaction wave, generated because of the overexpansion of explosive gases or reflections from the ground, catches with the shock front, causing a decay in the pressure profile. At a sufficiently longer distance from the explosion, a blast wave takes the form of the Friedlander wave^8^ (or so-called free-field blast wave). The free-field blast wave is characterized by an instantaneous rise in pressure followed by a non-linear decay. Broadly, the blast wave implied in BINT is a free-field blast wave referred to as a “primary blast” in BINT literature.

Given that the explosives are restricted, primary blast waves are typically generated in a laboratory using compressed gas-drive shock tubes .^9–15^ It has been shown, in these investigations, that shock tubes can generate primary blast waves in some form. Significant literature exists on the accurate generation (or lack thereof) of primary blast waves using shock tubes.^16–28^ The blast wave evolves considerably along the length of the shock tube,^17,18,21^ based on the design of the shock tube.^22,24,25,27^ It has also been demonstrated that the blast wave profile and specimen placement location critically affect biomechanical loading^16,23,28^ and injury outcome.^28^ It is difficult to generate similar blast wave profiles across various shock tubes. Thus, it is crucial to report the blast-related measurements rigorously. Further, as many measurements as possible should be made and care should be taken to avoid jet wind (occurring near the exit of the shock tube) and secondary loading effects on the specimen.^17,19–21,27^

There is a lack of standard protocol for the blast measurements (i.e., incident blast overpressure [BOP], reflected overpressure, and intracranial pressure [ICP]). In the literature, BOP has been measured and reported at varying distances from the specimen, with different types of sensors, and with various data acquisition rates.^9,11,13,20,29–32^ This has caused a large scatter in reported incident overpressures.^9,11,13,20,29–32^ As a result, correlating BOP with the biomechanical response (e.g., ICP) and comparing data across various investigations become challenging.^33^ We have collected and analyzed the experimental data from the literature across various models (head surrogates, post-mortem human subjects [PMHS], and rat).^33^ Our analysis (Supplementary Fig. S1) suggests that, for a given model, a reasonable correlation can be established between reflected overpressure measured on the surface of the head (generally at the point of initial impact location) and ICP measured in the brain. Hence, in addition to the measurement of BOP, it is advisable to measure reflected overpressures at a few locations on the surface of the head.

## Biomechanics of Blast-Induced Neurotrauma

Various mechanisms (direct transmission,^34–37^ skull flexure,^38–40^ thoracic,^3,41,42^ head acceleration,^43–46^ and cavitation,^32,47–50^) have been proposed for BINT. A large amount of literature (e.g., see previous works^51,52^ and references therein) exists on the dominance or lack of these mechanisms. Investigations in PMHS^53,54^ and computational human head models^33–35,37,39,55–58^ (CHHM) suggest the dominance of head transmission, with skull flexure accounting for high-frequency, low-amplitude oscillations in ICP response^39^ and localized pressure gradients.^38,40^ Thoracic mechanism was one of the earliest candidates^3^ of BINT; recent literature^59–61^ suggests that this mechanism may not be responsible for BINT. Compression of the thorax, however, can cause a sudden blood surge in the brain vasculature, leading to vascular pathology.^59,62^ Various researchers have observed either ameliorating or insignificant effects on animal pathology because of the shielding of the thorax.^63–68^ Well-designed protocols across the laboratories are probably needed to address the contribution of the thoracic mechanism to BINT.

Blast-induced head acceleration^43–46^ was observed as a dominant mechanism in several investigations using rodents. In these investigations, head acceleration alone led to diffuse axonal injury (DAI) pathology and memory deficits. Further, there is evidence of DAI in veterans with histories of blast exposure.^69–72^ Thus, there is a need to investigate the effect of blast-induced head acceleration and blast DAI pathology using primates, rodents, PMHS, and CHHM, given that it may offer significant new insights. Cerebrospinal fluid (CSF) cavitation was observed in investigations using PMHS^32^ and CHHM.^48–50^ However, additional experimental investigations explicitly focused on CSF cavitation, biomechanical cascade, and BINT are needed to corroborate these findings fully.

Injury thresholds for BINT are in the nascent stages. A few investigations^73,74^ suggest that incident BOP of ∼145 kPa is a threshold for mild BINT in rodents. Animal-to-human scaling^75^ has been attempted; however, robust thresholds for BINT in humans remain unavailable. Biofidelic, three-dimensional CHHM^34,35,37,39,55,57,58^ may eventually provide threshold values, provided significant clinical data and associated field measurements in humans become available. The use of blast dosimeter^76^ to characterize blast exposure in military and law enforcement personnel will be a promising step.

The role of helmets and goggles in blast protection is a critical issue. Numerous investigations suggest that these accessories only marginally mitigate or have an adverse effect under certain situations (e.g., a gap between the head and helmet,^40,77–79^ with visor and high-density foam pads,^80^ with better linking of the helmet with the head,^56^ excessive compression of foam pads).^81,82^ Particularly, negative ICPs and brain strains have been shown to increase with these accessories.^56,80^ Carefully constructed strategies will be required to address the challenges associated with personal protective equipment (PPE).

## Pathology of Blast-Induced Neurotrauma Using Animal Models

The pathology of BINT has been mainly studied using rodent models. Investigations, based on a single exposure, can be broadly categorized into mild (BOP, 85–145 kPa), moderate (BOP, 145–220 kPa), severe (BOP, >220 kPa), and fatal (>300 kPa).^83–87^ Fatal injuries represent animal mortality.^85,88–90^ For the BINT, blood–brain barrier (BBB) permeability and oxidative stress have been the most common pathological features. The degree of BBB permeability and oxidative stress depends on the intensity of the blast; these increase with an increase in the BOP.^91,92^ In cases of severe loading, BBB permeability is acute (i.e., manifesting almost instantaneously) and typically reversible in 3–4 days post-exposure.^91,93,94^ BBB permeability is subacute for mild loading, manifesting in 3–6 h post-exposure.^83,86,92,95,96^ BBB permeability is reversible, as evidenced by a significant decrease in extravasation observed beyond 24 h post-exposure.^83,86,92,95,96^ Numerous studies have noted both the acute and subacute phases for moderate loading.^84,97,98^ An investigation suggests that BBB permeability is not compromised below a BOP of 70 kPa.^99^ The most substantial changes attributable to the BBB and oxidative stress have been observed in the frontal cortex, striatum, thalamus, hippocampus, and amygdala.^95,98–102^ Apart from these peculiar pathological features, hemorrhage, edema, inflammation, DAI, vasospasm, neural loss, and metabolic changes have been observed for moderate and severe loading.^63,67,74,87,103–112^ Mild loading mainly manifests neural degeneration, astrocyte and microglial activation, DAI, multi-focal axonal damage, and microvascular damage.^43,64,83,101,113–123^

## Behavioral Outcomes of Blast-Induced Neurotrauma Using Animal Models

Most of the investigations of behavioral deficits are in rodents with BOPs in the range of 70–150 kPa.^43,66,90,124–135^ Anxiety,^101,136–138^ auditory^127,128,139–142^ and recognition memory deficits,^43,66,134,143–145^ and fear conditioning^129,132,133^ have been consistently reported behavioral deficits. These deficits were observed between 7 and 30 days post-exposure.^43,66,129,132–134,143,144^ Spatial working memory and motor deficits have also been reported, especially for animals kept outside the shock tube (and potentially subjected to the jet wind).^36,103,125,126,130,131,146^

## Repetitive Blast-Induced Neurotrauma Using Animal Models

Experimental investigations involving repetitive blast exposure are in the early stages, with most of the studies utilizing rodents exposed to BOPs ranging from 40 to 207 kPa.^29,95,121,132,147–162^ Reported pathology of repetitive BINT is very similar to the pathology observed during a single exposure.^95,121,147–158^ Various investigations have observed diverse neuropathological outcomes, including BBB permeability, oxidative stress, chronic vascular impairments, neurodegeneration, neuroinflammation, and axonal damage.^95,121,147–158,163^ The notable differences of repetitive BINT, with respect to a single exposure, include manifestation of BINT at lower BOPs (≥40 kPa) and chronic effects lasting for several months.^29,132,147^ Investigations also indicate heightened anxiety, motor impairments, and fear responses during subacute and chronic time points.^29,132,159–162,164–166^

## Clinical and Neuroimaging Investigations in Humans

Clinical studies have been conducted on combat veterans and law enforcement personnel to understand the effects of BINT. Human subjects have been exposed to multiple or repetitive blast.^167,168^ We could not find any investigation with a single exposure. Such a scenario is probably impractical in the combat theatre. Cerebral edema, intracranial hemorrhage, delayed vasospasm, perivascular damage, skull fracture, tissue damage, and intracranial hypertension have been observed in severely injured patients.^88,123,169–171^ In recent years, cases of mild BINT have increased.^172–175^ These studies have reported headaches, sleep disorder, poor motor skills, cognitive dysfunction, depression, and anxiety.^176–182^ These symptoms appear acutely and continue for months to years. Sustaining a mild BINT increases the likelihood of developing the risk of migraines, post-traumatic stress disorder, and neurocognitive impairment.^72,89,167,176,180,183–189^ Neuroimaging studies among military personnel have predominantly reported a loss of white matter integrity,^71,190–195^ and alterations in cortical volume and thickness.^196–204^ Other observed effects include DAI,^69–72^ alterations in the thalamic network,^205^ small cerebral microhemorrhages,^193^ and pituitary impairments.^193^

Delineating the effects of a primary blast from blunt and ballistic trauma in humans remains a paramount challenge.^72^ Recent investigations in special operations personnel, predominantly exposed to primary blast, are encouraging in this regard.^195,206–211^ These studies suggest that repetitive, low BOP (range, 7–90 kPa^207–209^) has the potential to cause neurological effects such as tinnitus, sleep imbalance, cognitive impairment, anxiety, insomnia, and headaches.^195,208–211^ Additional investigations (including longitudinal) in humans with a large sample size are needed to understand the short- and long-term effects of BINT. Such investigations will complement the laboratory studies.

In summary, reasonable progress has been made in each subdomain of BINT as discussed above. Pathology in rodents is most widely understood, followed by behavioral outcomes. Significant literature also exists on BINT mechanisms and the use of shock tubes for simulating primary blast waves. Clinical data in humans, exclusive to blast, are limited. Definitive injury thresholds for BINT in humans remain unavailable. Contemporary investigations of repetitive blast in targeted cohorts, exposed to primary blast, are encouraging. Future interdisciplinary research within and across various subdomains of BINT will probably lead to further progress and tangible outcomes.

## Supplementary Material

Supplemental data

## Transparency, Rigor, and Reproducibility Summary

This short communication is not a detailed review of various subdomains (Fig. 1) of BINT. Rather, it is a broad overview of the present state of knowledge in each subdomain. For a few subdomains, review articles^51,52,89,100,146,168,212,213^ are already available in the literature. Nonetheless, we have extensively gone through the literature in each subdomain. Relevant literature in each subdomain was considered while writing the manuscript; specifically, publications from 2000 to 2023 were included. Various search terms, such as “blast + rodent,” “blast + human,” “blast + imaging,” “blast + behavior,” “blast + TBI,” “blast + shock tube,” and “blast + biomechanical,” were used. The search items are a few sample search terms only. For blast wave generation and biomechanics subdomains, the corresponding author has been an active researcher for over a decade. Data collected and analyzed during this period were also considered during investigation and writing.

## Abbreviations Used

BINT: blast-induced neurotrauma
BOP: blast overpressure
CHHM: computational human head models
CSF: cerebrospinal fluid
DAI: diffuse axonal injury
ICP: intracranial pressure
PMHS: post-mortem human subjects
PPE: personal protective equipment

## References

[1] Tanielian T, Jaycox LH. Invisible wounds of war. RAND Corporation: Santa Monica, CA; 2008.

[2] Warden D. Military TBI during the Iraq and Afghanistan wars. J Head Trauma Rehabil 2006;21(5):398–402; doi: 10.1097/00001199-200609000-00004 16983225

[3] Cernak I, Wang ZG, Jiang JX, et al. Ultrastructural and functional characteristics of blast injury-induced neurotrauma. J Trauma 2001;50(4):695–706; doi: 10.1097/00005373-200104000-00017 11303167

[4] Cernak I, Wang ZG, Jiang JX, et al. Cognitive deficits following blast injury-induced neurotrauma: possible involvement of nitric oxide. Brain Inj 2001;15(7):593–612; doi: 10.1080/02699050010009559 11429089

[5] Trudeau DL, Anderson J, Hansen LM, et al. Findings of mild traumatic brain injury in combat veterans with PTSD and a history of blast concussion. J Neuropsychiatry Clin Neurosci 1998;10(3):308–313; doi: 10.1176/jnp.10.3.308 9706538

[6] Denny JW, Brown R, Head MG, et al. Allocation of funding into blast injury-related research and blast traumatic brain injury between 2000 and 2019: analysis of global investments from public and philanthropic funders. BMJ Mil Health 2023;169(2):127–132; doi: 10.1136/bmjmilitary-2020-001655 PMC10176327 33243763

[7] Kinney GF, Graham KJ. Explosive Shocks in Air. Springer-Verlag: New York, NY; 1985.

[8] Friedlander FG. The diffraction of sound pulses; diffraction by a semi-infinite plane. Proc R Soc Lond A Math Phys Sci 1946;186(1006):322–344; doi: 10.1098/rspa.1946.0046 20998736

[9] Chandra N, Sundaramurthy A, Gupta RK. Validation of laboratory animal and surrogate human models in primary blast injury studies. Mil Med 2017;182(S1):105–113; doi: 10.7205/milmed-d-16-00144 28291460

[10] Effgen GB, Hue CD, Vogel III E, et al. A multiscale approach to blast neurotrauma modeling: part II: methodology for inducing blast injury to in vitro models. Front Neurol 2012;3:23; doi: 10.3389/fneur.2012.00023 22375134 PMC3285773

[11] Long JB, Bentley TL, Wessner KA, et al. Blast overpressure in rats: recreating a battlefield injury in the laboratory. J Neurotrauma 2009;26(6):827–840; doi: 10.1089/neu.2008.0748 19397422

[12] Panzer MB, Matthews KA, Yu AW, et al. A multiscale approach to blast neurotrauma modeling: part I—development of novel test devices for in vivo and in vitro blast injury models. Front Neurol 2012;3:46; doi: 10.3389/fneur.2012.00046 22470367 PMC3314189

[13] Sawyer TW, Wang Y, Ritzel DV, et al. High-fidelity simulation of primary blast: direct effects on the head. J Neurotrauma 2016;33(13):1181–1193; doi: 10.1089/neu.2015.3914 26582146

[14] Kumar R, Nedungadi A. Using gas-driven shock tubes to produce blast wave signatures. Front Neurol 2020;11:90; doi: 10.3389/fneur.2020.00090 32153491 PMC7047208

[15] Reneer DV, Hisel RD, Hoffman JM, et al. A multi-mode shock tube for investigation of blast-induced traumatic brain injury. J Neurotrauma 2011;28(1):95–104; doi: 10.1089/neu.2010.1513 21083431 PMC3019584

[16] Alay E, Skotak M, Misistia A, et al. Dynamic loads on human and animal surrogates at different test locations in compressed-gas-driven shock tubes. Shock Waves 2018;28:51–62; doi: 10.1007/s00193-017-0762-4

[17] Chandra N, Ganpule S, Kleinschmit NN, et al. Evolution of blast wave profiles in simulated air blasts: experiment and computational modeling. Shock Waves 2012;22:403–415; doi: 10.1007/s00193-012-0399-2

[18] Chen Y, Constantini S. Caveats for using shock tube in blast-induced traumatic brain injury research. Front Neurol 2013;4:117; doi: 10.3389/fneur.2013.00117 23986741 PMC3752771

[19] Kahali S, Townsend M, Mendez Nguyen M, et al. The evolution of secondary flow phenomena and their effect on primary shock conditions in shock tubes: experimentation and numerical model. PLoS One 2020;15(1):e0227125; doi: 10.1371/journal.pone.0227125 31945083 PMC6964877

[20] Kuriakose M, Skotak M, Misistia A, et al. Tailoring the blast exposure conditions in the shock tube for generating pure, primary shock waves: the end plate facilitates elimination of secondary loading of the specimen. PLoS One 2016;11(9):e0161597; doi: 10.1371/journal.pone.0161597 27603017 PMC5014318

[21] Needham CE, Ritzel D, Rule GT, et al. Blast testing issues and TBI: experimental models that lead to wrong conclusions. Front Neurol 2015;6:72; doi: 10.3389/fneur.2015.00072 25904891 PMC4389725

[22] Nguyen TTN, Wilgeroth JM, Proud WG. Controlling blast wave generation in a shock tube for biological applications. J Phys Conf Ser 2014;500:142025; doi: 10.1088/1742-6596/500/14/142025

[23] Skotak M, Alay E, Zheng JQ, et al. Effective testing of personal protective equipment in blast loading conditions in shock tube: comparison of three different testing locations. PLoS One 2018;13:e0198968; doi: 10.1371/journal.pone.0198968 29894521 PMC5997325

[24] Sundaramurthy A, Chandra N. A parametric approach to shape field relevant blast wave profiles in compressed gas-driven shock tube. Front Neurol 2014;5:253; doi: 10.3389/fneur.2014.00253 25520701 PMC4251450

[25] Tasissa AF, Hautefeuille M, Fitek JH, et al. On the formation of Friedlander waves in a compressed-gas-driven shock tube. Proc Math Phys Eng Sci 2016;472(2186):20150611; doi: 10.1098/rspa.2015.0611 27118888 PMC4841653

[26] Yu AW, Bigler BR, Wood GW, et al. In vs. Out: Controversies in Shock Tube Blast Experiments. Mary Ann Liebert, Inc.: San Francisco, CA; 2014.

[27] Sutar S, Ganpule SG. Assessment of compression driven shock tube designs in replicating free-field blast conditions for traumatic brain injury studies. J Neurotrauma 2021;38(12):1717–1729; doi: 10.1089/neu.2020.7394 33108952

[28] Sundaramurthy A, Alai A, Ganpule S, et al. Blast-induced biomechanical loading of the rat: an experimental and anatomically accurate computational blast injury model. J Neurotrauma 2012;29(13):2352–2364; doi: 10.1089/neu.2012.2413 22620716

[29] Arun P, Wilder DM, Eken O, et al. Long-term effects of blast exposure: a functional study in rats using an advanced blast simulator. J Neurotrauma 2020;37(4):647–655; doi: 10.1089/neu.2019.6591 31595810

[30] Dal Cengio Leonardi A, Keane NJ, Bir CA, et al. Head orientation affects the intracranial pressure response resulting from shock wave loading in the rat. J Biomech 2012;45(15):2595–2602; doi: 10.1016/j.jbiomech.2012.08.024 22947434

[31] Iwaskiw A, Ott K, Armiger R, et al. The measurement of intracranial pressure and brain displacement due to short-duration dynamic overpressure loading. Shock Waves 2018;28(1):63–83; doi: 10.1007/s00193-017-0759-z

[32] Salzar RS, Treichler D, Wardlaw A, et al. Experimental investigation of cavitation as a possible damage mechanism in blast-induced traumatic brain injury in post-mortem human subject heads. J Neurotrauma 2017;34(8):1589–1602; doi: 10.1089/neu.2016.4600 27855566

[33] Sutar S, Ganpule S. Investigation of wave propagation through head layers with focus on understanding blast wave transmission. Biomech Model Mechanobiol 2020;19(3):875–892; doi: 10.1007/s10237-019-01256-9 31745681

[34] Ganpule S, Alai A, Plougonven E, et al. Mechanics of blast loading on the head models in the study of traumatic brain injury using experimental and computational approaches. Biomech Model Mechanobiol 2013;12(3):511–531; doi: 10.1007/s10237-012-0421-8 22832705

[35] Nyein MK, Jason AM, Yu L, et al. In silico investigation of intracranial blast mitigation with relevance to military traumatic brain injury. Proc Natl Acad Sci U S A 2010;107(48):20703–20708; doi: 10.1073/pnas.1014786107 21098257 PMC2996433

[36] Sundaramurthy A, Alai A, Ganpule S, et al. Blast-induced biomechanical loading of the rat: an experimental and anatomically accurate computational blast injury model. J Neurotrauma 2012;29(13):2352–2364; doi: 10.1089/neu.2012.2413 22620716

[37] Taylor PA, Ford CC. Simulation of blast-induced early-time intracranial wave physics leading to traumatic brain injury. J Biomech Eng 2009;131(6):061007; doi: 10.1115/1.3118765 19449961

[38] Bolander R, Mathie B, Bir C, et al. Skull flexure as a contributing factor in the mechanism of injury in the rat when exposed to a shock wave. Ann Biomed Eng 2011;39(10):2550–2559; doi: 10.1007/s10439-011-0343-0 21735320

[39] Garimella HT, Kraft RH, Przekwas AJ. Do blast induced skull flexures result in axonal deformation? PLoS One 2018;13(3):e0190881; doi: 10.1371/journal.pone.0190881 29547663 PMC5856259

[40] Moss WC, King MJ, Blackman EG. Skull flexure from blast waves: a mechanism for brain injury with implications for helmet design. Phys Rev Lett 2009;103(10):108702; doi: 10.1103/PhysRevLett.103.108702 19792349

[41] Courtney AC, Courtney MW. A thoracic mechanism of mild traumatic brain injury due to blast pressure waves. Med Hypotheses 2009;72(1):76–83; doi: 10.1016/j.mehy.2008.08.015 18829180

[42] Koliatsos VE, Cernak I, Xu L, et al. A mouse model of blast injury to brain: initial pathological, neuropathological, and behavioral characterization. J Neuropathol Exp Neurol 2011;70(5):399–416; doi: 10.1097/NEN.0b013e3182189f06 21487304

[43] Goldstein LE, Fisher AM, Tagge CA, et al. Chronic traumatic encephalopathy in blast-exposed military veterans and a blast neurotrauma mouse model. Sci Transl Med 2012;4(134):134ra60; doi: 10.1126/scitranslmed.3003716 PMC3739428 22593173

[44] Risling M, Plantman S, Angeria M, et al. Mechanisms of blast induced brain injuries, experimental studies in rats. Neuroimage 2011;54 Suppl 1:S89–S97; doi: 10.1016/j.neuroimage.2010.05.031 20493951

[45] Aravind A, Kosty J, Chandra N, et al. Blast exposure predisposes the brain to increased neurological deficits in a model of blast plus blunt traumatic brain injury. Exp Neurol 2020;332:113378; doi: 10.1016/j.expneurol.2020.113378 32553593

[46] Gullotti DM, Beamer M, Panzer MB, et al. Significant head accelerations can influence immediate neurological impairments in a murine model of blast-induced traumatic brain injury. J Biomech Eng 2014;136(9):091004; doi: 10.1115/1.4027873 24950710

[47] Goeller J, Wardlaw A, Treichler D, et al. Investigation of cavitation as a possible damage mechanism in blast-induced traumatic brain injury. J Neurotrauma 2012;29(10):1970–1981, doi: 10.1089/neu.2011.2224 22489674

[48] Haniff S, Taylor PA. In silico investigation of blast-induced intracranial fluid cavitation as it potentially leads to traumatic brain injury. Shock Waves 2017;27(6):929–945; doi: 10.1007/s00193-017-0765-1

[49] Panzer MB, Myers BS, Capehart BP, et al. Development of a finite element model for blast brain injury and the effects of CSF cavitation. Ann Biomed Eng 2012;40(7):1530–1544; doi: 10.1007/s10439-012-0519-2 22298329

[50] Yu X, Azor A, Sharp DJ, et al. Mechanisms of tensile failure of cerebrospinal fluid in blast traumatic brain injury. Extreme Mechanics Letters 2020;38:100739; doi: 10.1016/j.eml.2020.100739

[51] Fievisohn E, Bailey Z, Guettler A, et al. Primary blast brain injury mechanisms: current knowledge, limitations, and future directions. J Biomech Eng 2018;140(2):020806; doi: 10.1115/1.4038710. 29222564

[52] Courtney A, Courtney M. The complexity of biomechanics causing primary blast-induced traumatic brain injury: a review of potential mechanisms. Front Neurol 2015;6:221; doi: 10.3389/fneur.2015.00221 26539158 PMC4609847

[53] Bir C. Measuring blast-related intracranial pressure within the human head. Final report. U.S. Army Medical Research and Materiel Command Award Number W81XWH-09-1-0498. 2011.

[54] Ganpule S, Salzar R, Perry B, et al. Role of helmets in blast mitigation: insights from experiments on PMHS surrogate. Int J Exp Comput Biomech 2016;4(1):13–31; doi: 10.1504/IJECB.2016.081745

[55] Chafi MS, Karami G, Ziejewski M. Biomechanical assessment of brain dynamic responses due to blast pressure waves. Ann Biomed Eng 2010;38(2):490–504; doi: 10.1007/s10439-009-9813-z 19806456

[56] Yu X, Ghajari M. Protective performance of helmets and goggles in mitigating brain biomechanical response to primary blast exposure. Ann Biomed Eng 2022 Nov;50(11):1579–1595; doi: 10.1007/s10439-022-02936-x 35296943 PMC9652178

[57] Zhang L, Makwana R, Sharma S. Brain response to primary blast wave using validated finite element models of human head and advanced combat helmet. Front Neurol 2013;4:88; doi: 10.3389/fneur.2013.00088 23935591 PMC3731672

[58] Sutar S, Ganpule S. Evaluation of blast simulation methods for modeling blast wave interaction with human head. J Biomech Eng 2021;144(5):051009; doi: 10.1115/1.4053059 34791052

[59] Rubio JE, Skotak M, Alay E, et al. Does blast exposure to the torso cause a blood surge to the brain? Front Bioeng Biotechnol 2020;8:573647; doi: 10.3389/fbioe.2020.573647 33392161 PMC7773947

[60] Rubio JE, Unnikrishnan G, Sajja VSSS, et al. Investigation of the direct and indirect mechanisms of primary blast insult to the brain. Sci Rep 2021;11(1):16040; doi: 10.1038/s41598-021-95003-9 34362935 PMC8346555

[61] Sajja V, Shoge R, McNeil E, et al. Comparison of biomechanical outcome measures from characteristically different blast simulators and the influence of exposure location. Mil Med 2023;188(Suppl 6):288–294; doi: 10.1093/milmed/usad111 37948259

[62] Assari S, Laksari K, Barbe M, et al. Cerebral Blood Pressure Rise During Blast Exposure in a Rat Model of Blast-Induced Traumatic Brain Injury. American Society of Mechanical Engineers: New York, NY; 2013.

[63] Koliatsos VE, Cernak I, Xu L, et al. A mouse model of blast injury to brain: initial pathological, neuropathological, and behavioral characterization. J Neuropathol Exp Neurol 2011;70(5):399–416; doi: 10.1097/NEN.0b013e3182189f06 21487304

[64] Xu L, Schaefer ML, Linville RM, et al. Neuroinflammation in primary blast neurotrauma: time course and prevention by torso shielding. Exp Neurol 2016;277:268–274; doi: 10.1016/j.expneurol.2016.01.010 26784004

[65] Cernak I. The importance of systemic response in the pathobiology of blast-induced neurotrauma. Front Neurol 2010;1:151; doi: 10.3389/fneur.2010.00151 21206523 PMC3009449

[66] Long JB, Bentley TL, Wessner KA, et al. Blast overpressure in rats: recreating a battlefield injury in the laboratory. J Neurotrauma 2009;26(6):827–840; doi: 10.1089/neu.2008.0748 19397422

[67] Tompkins P, Tesiram Y, Lerner M, et al. Brain injury: neuro-inflammation, cognitive deficit, and magnetic resonance imaging in a model of blast induced traumatic brain injury. J Neurotrauma 2013;30(22):1888–1897; doi: 10.1089/neu.2012.2674 23777197

[68] Säljö A, Arrhén F, Bolouri H, et al. Neuropathology and pressure in the pig brain resulting from low-impulse noise exposure. J Neurotrauma 2008;25(12):1397–1406; doi: 10.1089/neu.2008.0602 19146459

[69] Ryu J, Horkayne-Szakaly I, Xu L, et al. The problem of axonal injury in the brains of veterans with histories of blast exposure. Acta Neuropathol Commun 2014;2:153; doi: 10.1186/s40478-014-0153-3 25422066 PMC4260204

[70] Vakhtin AA, Calhoun VD, Jung RE, et al. Changes in intrinsic functional brain networks following blast-induced mild traumatic brain injury. Brain Inj 2013;27(11):1304–1310; doi: 10.3109/02699052.2013.823561 24020442 PMC5075489

[71] Mac Donald CL, Johnson AM, Cooper D, et al. Detection of blast-related traumatic brain injury in US military personnel. N Engl J Med 2011;364(22):2091–2100; doi: 10.1056/NEJMoa1008069 21631321 PMC3146351

[72] Phipps H, Mondello S, Wilson A, et al. Characteristics and impact of US military blast-related mild traumatic brain injury: a systematic review. Front Neurol 2020;11:559318; doi: 10.3389/fneur.2020.559318 33224086 PMC7667277

[73] Mishra V, Skotak M, Schuetz H, et al. Primary blast causes mild, moderate, severe and lethal TBI with increasing blast overpressures: experimental rat injury model. Sci Rep 2016;6:26992; doi: 10.1038/srep26992 27270403 PMC4895217

[74] Rafaels KA, Cameron R, Panzer MB, et al. Brain injury risk from primary blast. J Trauma Acute Care Surg 2012;73(4):895–901; doi: 10.1097/TA.0b013e31825a760e 22836001

[75] Jean A, Nyein MK, Zheng JQ, et al. An animal-to-human scaling law for blast-induced traumatic brain injury risk assessment. Proc Natl Acad Sci U S A 2014;111(43):15310–15315; doi: 10.1073/pnas.1415743111 25267617 PMC4217421

[76] Borkholder D. Concept to Commercialization of a MEMS-based Blast Dosimetry System. IEEE: New York, NY; 2015.

[77] Mott D, Schwer D, Young TR, et al. Blast-induced pressure fields beneath a military helmet. Proceedings of the American Physical Society, 61st Annual Meeting of the APS Division of Fluid Dynamics, November 23–25, 2008, abstract id. MF.008.

[78] Ganpule S, Gu L, Alai A, et al. Role of helmet in the mechanics of shock wave propagation under blast loading conditions. Comput Methods Biomech Biomed Engin 2012;15(11):1233–1244; doi: 10.1080/10255842.2011.597353 21806412

[79] Li J, Ma T, Huang C, et al. Protective mechanism of helmet under far-field shock wave. Int J Impact Eng 2020;143:103617; doi 10.1016/j.ijimpeng.2020.103617

[80] Singh D, Cronin D. Efficacy of visor and helmet for blast protection assessed using a computational head model. Shock Waves 2017;27(6):905–918; doi: 10.1007/s00193-017-0732-x

[81] Zhang TG, Fulton A, Ravi-Chandar K, et al. Rate Dependent Material Model for Helmet Pads. American Society of Mechanical Engineers: New York, NY; 2020.

[82] Zhang TG, Satapathy SS. Effect of Helmet Pads on the Load Transfer to Head Under Blast Loadings. American Society of Mechanical Engineers: New York, NY; 2014.

[83] Abdul-Muneer P, Schuetz H, Wang F, et al. Induction of oxidative and nitrosative damage leads to cerebrovascular inflammation in an animal model of mild traumatic brain injury induced by primary blast. Free Radic Biol Med 2013;60:282–291; doi: 10.1016/j.freeradbiomed.2013.02.029 23466554 PMC4007171

[84] Kuriakose M, Younger D, Ravula AR, et al. Synergistic role of oxidative stress and blood-brain barrier permeability as injury mechanisms in the acute pathophysiology of blast-induced neurotrauma. Sci Rep 2019;9(1):7717; doi: 10.1038/s41598-019-44147-w 31118451 PMC6531444

[85] Mishra V, Skotak M, Schuetz H, et al. Primary blast causes mild, moderate, severe and lethal TBI with increasing blast overpressures: Experimental rat injury model. Sci Rep 2016;6:26992; doi: 10.1038/srep26992 27270403 PMC4895217

[86] Skotak M, Wang F, Alai A, et al. Rat injury model under controlled field-relevant primary blast conditions: acute response to a wide range of peak overpressures. J Neurotrauma 2013;30(13):1147–1160; doi: 10.1089/neu.2012.2652 23362798 PMC3700437

[87] Turner RC, Naser ZJ, Logsdon AF, et al. Modeling clinically relevant blast parameters based on scaling principles produces functional & histological deficits in rats. Exp Neurol 2013;248:520–529; doi: 10.1016/j.expneurol.2013.07.008 23876514 PMC13418175

[88] Colamaria A, Blagia M, Carbone F, et al. Blast-related traumatic brain injury: report of a severe case and review of the literature. Surg Neurol Int 2022;13:151; doi: 10.25259/SNI_1134_2021 35509563 PMC9062926

[89] Rosenfeld JV, McFarlane AC, Bragge P, et al. Blast-related traumatic brain injury. Lancet Neurol 2013;12(9):882–893; doi: 10.1016/S1474-4422(13)70161-3 23884075

[90] Kobeissy F, Mondello S, Tümer N, et al. Assessing neuro-systemic & behavioral components in the pathophysiology of blast-related brain injury. Front Neurol 2013;4:186; doi: doi: 10.3389/fneur.2013.00186 24312074 PMC3836009

[91] Yeoh S, Bell ED, Monson KL. Distribution of blood–brain barrier disruption in primary blast injury. Ann Biomed Eng 2013;41:2206–2214; doi: 10.1007/s10439-013-0805-7 23568152

[92] Kabu S, Jaffer H, Petro M, et al. Blast-associated shock waves result in increased brain vascular leakage and elevated ROS levels in a rat model of traumatic brain injury. PLoS One 2015;10(5):e0127971; doi: 10.1371/journal.pone.0127971 26024446 PMC4449023

[93] Hue CD, Cho FS, Cao S, et al. Time course and size of blood–brain barrier opening in a mouse model of blast-induced traumatic brain injury. J Neurotrauma 2016;33(13):1202–1211; doi; 10.1089/neu.2015.4067 26414212

[94] Hue CD, Cao S, Haider SF, et al. Blood-brain barrier dysfunction after primary blast injury in vitro. J Neurotrauma 2013;30(19):1652–1663; doi: 10.1089/neu.2012.2773 23581482

[95] Kawoos U, Gu M, Lankasky J, et al. Effects of exposure to blast overpressure on intracranial pressure and blood-brain barrier permeability in a rat model. PLoS One 2016;11(12):e0167510; doi: 10.1371/journal.pone.0167510 27907158 PMC5132256

[96] Readnower RD, Chavko M, Adeeb S, et al. Increase in blood–brain barrier permeability, oxidative stress, and activated microglia in a rat model of blast-induced traumatic brain injury. J Neurosci Res 2010;88(16):3530–3539; doi: 10.1002/jnr.22510 20882564 PMC2965798

[97] Liu M, Zhang C, Liu W, et al. A novel rat model of blast-induced traumatic brain injury simulating different damage degree: implications for morphological, neurological, and biomarker changes. Front Cell Neurosci 2015;9:168; doi: 10.3389/fncel.2015.00168 25983677 PMC4416450

[98] Rama Rao KV, Iring S, Younger D, et al. A single primary blast-induced traumatic brain injury in a rodent model causes cell-type dependent increase in nicotinamide adenine dinucleotide phosphate oxidase isoforms in vulnerable brain regions. J Neurotrauma 2018;35(17):2077–2090; doi: 10.1089/neu.2017.5358 29648986 PMC6098412

[99] Kuriakose M, Rama Rao KV, Younger D, et al. Temporal and spatial effects of blast overpressure on blood-brain barrier permeability in traumatic brain injury. Sci Rep 2018;8(1):8681; doi: 10.1038/s41598-018-26813-7 29875451 PMC5989233

[100] Elder GA, Gama Sosa MA, De Gasperi R, et al. Vascular and inflammatory factors in the pathophysiology of blast-induced brain injury. Front Neurol 2015;6:48; doi; 10.3389/fneur.2015.00048 25852632 10.3389/fneur.2015.00048 PMC4360816

[101] Sajja VS, Hubbard WB, VandeVord PJ. Subacute oxidative stress and glial reactivity in the amygdala are associated with increased anxiety following blast neurotrauma. Shock 2015;44:71–78; doi: 10.1097/SHK.0000000000000311 25521536

[102] Kochanek PM, Dixon CE, Shellington DK, et al. Screening of biochemical and molecular mechanisms of secondary injury and repair in the brain after experimental blast-induced traumatic brain injury in rats. J Neurotrauma 2013;30(11):920–937; doi: 10.1089/neu.2013.2862 23496248 PMC5586163

[103] Svetlov SI, Prima V, Kirk DR, et al. Morphologic and biochemical characterization of brain injury in a model of controlled blast overpressure exposure. J Trauma Acute Care Surg 2010;69(4):795–804; doi: 10.1097/TA.0b013e3181bbd885 20215974

[104] Cheng J, Gu J, Ma Y, et al. Development of a rat model for studying blast-induced traumatic brain injury. J Neurol Sci 2010;294(1-2):23–28; doi: 10.1016/j.jns.2010.04.010 20478573

[105] Kuehn R, Simard PF, Driscoll I, et al. Rodent model of direct cranial blast injury. J Neurotrauma 2011;28(10):2155–2169; doi: 10.1089/neu.2010.1532 21639724

[106] Ahlers ST, Vasserman-Stokes E, Shaughness MC, et al. Assessment of the effects of acute and repeated exposure to blast overpressure in rodents: toward a greater understanding of blast and the potential ramifications for injury in humans exposed to blast. Front Neurol 2012;3:32; doi: 10.3389/fneur.2012.00032 22403572 PMC3293241

[107] Reneer DV, Hisel RD, Hoffman JM, et al. A multi-mode shock tube for investigation of blast-induced traumatic brain injury. J Neurotrauma 2011;28(1):95–104; doi: 10.1089/neu.2010.1513 21083431 PMC3019584

[108] Garman RH, Jenkins LW, Switzer RC III, et al. Blast exposure in rats with body shielding is characterized primarily by diffuse axonal injury. J Neurotrauma 2011;28(6):947–959; doi: 10.1089/neu.2010.1540 21449683

[109] Risling M, Plantman S, Angeria M, et al. Mechanisms of blast induced brain injuries, experimental studies in rats. Neuroimage 2011;54 Suppl 1:S89–S97; doi: 10.1016/j.neuroimage.2010.05.031 20493951

[110] Bauman RA, Ling G, Tong L, et al. An introductory characterization of a combat-casualty-care relevant swine model of closed head injury resulting from exposure to explosive blast. J Neurotrauma 2009;26(6):841–860; doi: 10.1089/neu.2008.0898 19215189

[111] de Lanerolle NC, Bandak F, Kang D, et al. Characteristics of an explosive blast-induced brain injury in an experimental model. J Neuropathol Exp Neurol 2011;70(11):1046–1057; doi: 10.1097/NEN.0b013e318235bef2 22002430

[112] Cao R, Zhang C, Mitkin VV, et al. Comprehensive characterization of cerebrovascular dysfunction in blast traumatic brain injury using photoacoustic microscopy. J Neurotrauma 2019;36(10):1526–1534; doi: 10.1089/neu.2018.6062 30501547 PMC6532277

[113] Rodriguez UA, Zeng Y, Deyo D, et al. Effects of mild blast traumatic brain injury on cerebral vascular, histopathological, and behavioral outcomes in rats. J Neurotrauma 2018;35(2):375–392; doi: 10.1089/neu.2017.5256 29160141 PMC5784797

[114] Abutarboush R, Gu M, Kawoos U, et al. Exposure to blast overpressure impairs cerebral microvascular responses and alters vascular and astrocytic structure. J Neurotrauma 2019;36(22):3138–3157; doi: 10.1089/neu.2019.6423 31210096 PMC6818492

[115] Clark AT, Abrahamson EE, Harper MM, et al. Chronic effects of blast injury on the microvasculature in a transgenic mouse model of Alzheimer's disease related Aβ amyloidosis. Fluids Barriers CNS 2022;19(1):5; doi: 10.1186/s12987-021-00301-z 35012589 PMC8751260

[116] Cho HJ, Sajja VS, Vandevord PJ, et al. Blast induces oxidative stress, inflammation, neuronal loss and subsequent short-term memory impairment in rats. Neuroscience 2013;253:9–20; doi: 10.1016/j.neuroscience.2013.08.037 23999126

[117] Hernandez A, Tan C, Plattner F, et al. Exposure to mild blast forces induces neuropathological effects, neurophysiological deficits and biochemical changes. Mol Brain 2018;11(1):64; doi: 10.1186/s13041-018-0408-1 30409147 PMC6225689

[118] Shetty AK, Mishra V, Kodali M, et al. Blood brain barrier dysfunction and delayed neurological deficits in mild traumatic brain injury induced by blast shock waves. Front Cell Neurosci 2014;8:232; doi: 10.3389/fncel.2014.00232 25165433 PMC4131244

[119] Sato S, Kawauchi S, Okuda W, et al. Real-time optical diagnosis of the rat brain exposed to a laser-induced shock wave: observation of spreading depolarization, vasoconstriction and hypoxemia-oligemia. PLoS One 2014;9(1):e82891; doi: 10.1371/journal.pone.0082891 24416150 PMC3885400

[120] Kwon S-KC, Kovesdi E, Gyorgy AB, et al. Stress and traumatic brain injury: a behavioral, proteomics, and histological study. Front Neurol 2011;2:12; doi: 10.3389/fneur.2011.00012 21441982 PMC3057553

[121] Gama Sosa MA, De Gasperi R, Janssen PL, et al. Selective vulnerability of the cerebral vasculature to blast injury in a rat model of mild traumatic brain injury. Acta Neuropathol Commun 2014;2:67; doi: 10.1186/2051-5960-2-67 24938728 PMC4229875

[122] Pun PB, Kan EM, Salim A, et al. Low level primary blast injury in rodent brain. Front Neurol 2011;2:19; doi: 10.3389/fneur.2011.00019 21541261 PMC3083909

[123] Agoston DV, Elsayed M. Serum-based protein biomarkers in blast-induced traumatic brain injury spectrum disorder. Front Neurol 2012;3:107; doi: 10.3389/fneur.2012.00107 22783223 PMC3390892

[124] Antunes M, Biala G. The novel object recognition memory: neurobiology, test procedure, and its modifications. Cogn Process 2012;13(2):93–110; doi: 10.1007/s10339-011-0430-z 22160349 PMC3332351

[125] Buitrago MM, Schulz JB, Dichgans J, et al. Short and long-term motor skill learning in an accelerated rotarod training paradigm. Neurobiol Learn Mem 2004;81(3):211–216; doi: 10.1016/j.nlm.2004.01.001 15082022

[126] Elder GA, Dorr NP, De Gasperi R, et al. Blast exposure induces post-traumatic stress disorder-related traits in a rat model of mild traumatic brain injury. J Neurotrauma 2012;29(16):2564–2575; doi: 10.1089/neu.2012.2510 22780833 PMC3495123

[127] Luo H, Pace E, Zhang X, et al. Blast-Induced tinnitus and spontaneous firing changes in the rat dorsal cochlear nucleus. J Neurosci Res 2014;92(11):1466–1477; doi: 10.1002/jnr.23424 24938852

[128] Mao JC, Pace E, Pierozynski P, et al. Blast-induced tinnitus and hearing loss in rats: behavioral and imaging assays. J Neurotrauma 2012;29(2):430–444; doi: 10.1089/neu.2011.1934 21933015 PMC3261792

[129] McDannald MA. Contributions of the amygdala central nucleus and ventrolateral periaqueductal grey to freezing and instrumental suppression in Pavlovian fear conditioning. Behav Brain Res 2010;211(1):111–117; doi: 10.1016/j.bbr.2010.03.020 20298722 PMC2862132

[130] Mizoguchi K, Yuzurihara M, Ishige A, et al. Chronic stress impairs rotarod performance in rats: implications for depressive state. Pharmacol Biochem Behav 2002;71(1–2):79–84; doi: 10.1016/s0091-3057(01)00636-0 11812510

[131] Park E, Eisen R, Kinio A, et al. Electrophysiological white matter dysfunction and association with neurobehavioral deficits following low-level primary blast trauma. Neurobiol Dis 2013;52:150–159; doi: 10.1016/j.nbd.2012.12.002 23238347

[132] Perez-Garcia G, Gama Sosa MA, De Gasperi R, et al. Chronic post-traumatic stress disorder-related traits in a rat model of low-level blast exposure. Behav Brain Res 2018;340:117–125; doi: 10.1016/j.bbr.2016.09.061 27693852 PMC11181290

[133] Pickens CL, Navarre BM, Nair SG. Incubation of conditioned fear in the conditioned suppression model in rats: role of food-restriction conditions, length of conditioned stimulus, and generality to conditioned freezing. Neuroscience 2010;169(4):1501–1510; doi: 10.1016/j.neuroscience.2010.06.036 20600654 PMC2920731

[134] Rubovitch V, Ten-Bosch M, Zohar O, et al. A mouse model of blast-induced mild traumatic brain injury. Exp Neurol 2011;232(2):280–289; doi: 10.1016/j.expneurol.2011.09.018 21946269 PMC3202080

[135] Cernak I, Merkle AC, Koliatsos VE, et al. The pathobiology of blast injuries and blast-induced neurotrauma as identified using a new experimental model of injury in mice. Neurobiol Dis 2011;41(2):538–551; doi: 10.1016/j.nbd.2010.10.025 21074615

[136] Awwad HO, Gonzalez LP, Tompkins P, et al. Blast overpressure waves induce transient anxiety and regional changes in cerebral glucose metabolism and delayed hyperarousal in rats. Front Neurol 2015;6:132; doi: 10.3389/fneur.2015.00132 26136722 PMC4470265

[137] Kamnaksh A, Kovesdi E, Kwon SK, et al. Factors affecting blast traumatic brain injury. J Neurotrauma 2011;28(10):2145–2153; doi: 10.1089/neu.2011.1983 21861635

[138] Russell AL, Handa RJ, Wu TJ. Sex-dependent effects of mild blast-induced traumatic brain injury on corticotropin-releasing factor receptor gene expression: potential link to anxiety-like behaviors. Neuroscience 2018;392:1–12; doi: 10.1016/j.neuroscience.2018.09.014 30248435

[139] Oleksiak M, Smith BM, St Andre JR, et al. Audiological issues and hearing loss among Veterans with mild traumatic brain injury. J Rehabil Res Dev 2012;49(7):995–1004. doi: 10.1682/jrrd.2011.01.0001 23341275

[140] Swan A, Nelson J, Swiger B, et al. Prevalence of hearing loss and tinnitus in Iraq and Afghanistan Veterans: a Chronic Effects of Neurotrauma Consortium study. Hear Res 2017;349:4–12; doi: 10.1016/j.heares.2017.01.013 28153668

[141] Yurgil KA, Clifford RE, Risbrough VB, et al. Prospective associations between traumatic brain injury and postdeployment tinnitus in active-duty marines. J Head Trauma Rehabil 2016;31(1):30–39; doi: 10.1097/HTR.0000000000000117 25699623

[142] Galazyuk A, Hébert S. Gap-prepulse inhibition of the acoustic startle reflex (GPIAS) for tinnitus assessment: current status and future directions. Front Neurol 2015;6:88; doi: 10.3389/fneur.2015.00088 25972836 PMC4411996

[143] Song H, Konan LM, Cui J, et al. Ultrastructural brain abnormalities and associated behavioral changes in mice after low-intensity blast exposure. Behav Brain Res 2018;347:148–157; doi: 10.1016/j.bbr.2018.03.007 29526786

[144] Stemper BD, Shah AS, Budde MD, et al. Behavioral outcomes differ between rotational acceleration and blast mechanisms of mild traumatic brain injury. Front Neurol 2016;7:31; doi: 10.3389/fneur.2016.00031 27014184 PMC4789366

[145] Beamer M, Tummala SR, Gullotti D, et al. Primary blast injury causes cognitive impairments and hippocampal circuit alterations. Exp Neurol 2016;283(Pt A):16–28; doi: 10.1016/j.expneurol.2016.05.025 27246999 PMC5062598

[146] Aravind A, Ravula AR, Chandra N, et al. Behavioral deficits in animal models of blast traumatic brain injury. Front Neurol 2020;11:990; doi: 10.3389/fneur.2020.00990 33013653 PMC7500138

[147] Gama Sosa MA, De Gasperi R, Pryor D, et al. Late chronic local inflammation, synaptic alterations, vascular remodeling and arteriovenous malformations in the brains of male rats exposed to repetitive low-level blast overpressures. Acta Neuropathol Commun 2023;11(1):81; doi: 10.1186/s40478-023-01553-6 37173747 PMC10176873

[148] Hubbard WB, Vekaria HJ, Velmurugan GV, et al. Mitochondrial dysfunction after repeated mild blast traumatic brain injury is attenuated by a mild mitochondrial uncoupling prodrug. J Neurotrauma 2023;40(21–22):2396–2409; doi: 10.1089/neu.2023.0102 37476976 PMC10653072

[149] Dickstein DL, De Gasperi R, Gama Sosa MA, et al. Brain and blood biomarkers of tauopathy and neuronal injury in humans and rats with neurobehavioral syndromes following blast exposure. Mol Psychiatry 2021;26(10):5940–5954; doi: 10.1038/s41380-020-0674-z 32094584 PMC7484380

[150] Bradshaw Jr DV, Kim Y, Fu A, et al. Repetitive blast exposure produces white matter axon damage without subsequent myelin remodeling: in vivo analysis of brain injury using fluorescent reporter mice. Neurotrauma Rep 2021;2(1):180–192; doi: 10.1089/neur.2020.0058 34013219 PMC8127063

[151] Heyburn L, Abutarboush R, Goodrich S, et al. Repeated low-level blast acutely alters brain cytokines, neurovascular proteins, mechanotransduction, and neurodegenerative markers in a rat model. Front Cell Neurosci 2021;15:636707; doi: 10.3389/fncel.2021.636707 33679327 PMC7933446

[152] Heyburn L, Batuure A, Wilder D, et al. Neuroinflammation profiling of brain cytokines following repeated blast exposure. Int J Mol Sci 2023;24(16):12564; doi: 10.3390/ijms241612564 37628746 PMC10454588

[153] Arun P, Rittase WB, Wilder DM, et al. Defective methionine metabolism in the brain after repeated blast exposures might contribute to increased oxidative stress. Neurochem Int 2018;112:234–238; doi: 10.1016/j.neuint.2017.07.014 28774719

[154] Bittar A, Bhatt N, Hasan TF, et al. Neurotoxic tau oligomers after single versus repetitive mild traumatic brain injury. Brain Commun 2019;1(1):fcz004; doi: 10.1093/braincomms/fcz004 31608324 PMC6777515

[155] Huber BR, Meabon JS, Hoffer ZS, et al. Blast exposure causes dynamic microglial/macrophage responses and microdomains of brain microvessel dysfunction. Neuroscience 2016;319:206–220; doi: 10.1016/j.neuroscience.2016.01.022 26777891 PMC5274718

[156] Logsdon AF, Meabon JS, Cline MM, et al. Blast exposure elicits blood-brain barrier disruption and repair mediated by tight junction integrity and nitric oxide dependent processes. Sci Rep 2018;8(1):11344; doi: 10.1038/s41598-018-29341-6 30054495 PMC6063850

[157] Uzunalli G, Herr S, Dieterly AM, et al. Structural disruption of the blood–brain barrier in repetitive primary blast injury. Fluids Barriers CNS 2021;18(1):2; doi: 10.1186/s12987-020-00231-2 33413513 PMC7789532

[158] Toklu HZ, Yang Z, Oktay S, et al. Overpressure blast injury-induced oxidative stress and neuroinflammation response in rat frontal cortex and cerebellum. Behav Brain Res 2018;340:14–22; doi: 10.1016/j.bbr.2017.04.025 28419850

[159] Perez Garcia G, Perez GM, De Gasperi R, et al. Progressive cognitive and post-traumatic stress disorder-related behavioral traits in rats exposed to repetitive low-level blast. J Neurotrauma 2021;38(14):2030–2045; doi: 10.1089/neu.2020.7398 33115338 PMC8418528

[160] Hall AA, Mendoza MI, Zhou H, et al. Repeated low intensity blast exposure is associated with damaged endothelial glycocalyx and downstream behavioral deficits. Front Behav Neurosci 2017;11:104; doi: 10.3389/fnbeh.2017.00104 28649193 PMC5465256

[161] Blaze J, Choi I, Wang Z, et al. Blast-related mild TBI alters anxiety-like behavior and transcriptional signatures in the rat amygdala. Front Behav Neurosci 2020;14:160; doi: 10.3389/fnbeh.2020.00160 33192359 PMC7604767

[162] Ravula AR, Rodriguez J, Younger D, et al. Animal model of repeated low-level blast traumatic brain injury displays acute and chronic neurobehavioral and neuropathological changes. Exp Neurol 2022;349:113938; doi: 10.1016/j.expneurol.2021.113938 34863680

[163] Hue CD, Cao S, Dale Bass CR, et al. Repeated primary blast injury causes delayed recovery, but not additive disruption, in an in vitro blood–brain barrier model. J Neurotrauma 2014;31(10):951–960; doi: 10.1089/neu.2013.3149 24372353

[164] Perez-Garcia G, De Gasperi R, Sosa MAG, et al. PTSD-related behavioral traits in a rat model of blast-induced mTBI are reversed by the mGluR2/3 receptor antagonist BCI-838. eNeuro 2018;5(1):ENEURO.0357-17.2018; doi: 10.1523/ENEURO.0357-17.2018 PMC5790754 29387781

[165] Perez-Garcia G, Gama Sosa MA, De Gasperi R, et al. Exposure to a predator scent induces chronic behavioral changes in rats previously exposed to low-level blast: implications for the relationship of blast-related TBI to PTSD. Front Neurol 2016;7:176; doi: 10.3389/fneur.2016.00176 27803688 PMC5067529

[166] Miyai K, Kawauchi S, Kato T, et al. Axonal damage and behavioral deficits in rats with repetitive exposure of the brain to laser-induced shock waves: effects of inter-exposure time. Neurosci Lett 2021;749:135722; doi: 10.1016/j.neulet.2021.135722 33592306

[167] Belding JN, Kolaja CA, Rull RP, et al. Single and repeated high-level blast, low-level blast, and new-onset self-reported health conditions in the US Millennium Cohort Study: an exploratory investigation. Front Neurol 2023;14:1110717; doi: 10.3389/fneur.2023.1110717 37025202 PMC10070873

[168] Belding JN, Englert RM, Fitzmaurice S, et al. Potential health and performance effects of high-level and low-level blast: a scoping review of two decades of research. Front Neurol 2021;12:628782; doi: 10.3389/fneur.2021.628782 33776888 PMC7987950

[169] Armonda RA, Bell RS, Vo AH, et al. Wartime traumatic cerebral vasospasm: recent review of combat casualties. Neurosurgery 2006;59(6):1215–1225; discussion, 1225; doi: 10.1227/01.NEU.0000249190.46033.94 17277684

[170] Magnuson J, Leonessa F, Ling GS. Neuropathology of explosive blast traumatic brain injury. Curr Neurol Neurosci Rep 2012;12:570–579; doi: 10.1007/s11910-012-0303-6 22836523

[171] Nakagawa A, Manley GT, Gean AD, et al. Mechanisms of primary blast-induced traumatic brain injury: insights from shock-wave research. J Neurotrauma 2011;28(6):1101–1119; doi: 10.1089/neu.2010.1442 21332411

[172] Hoge CW, McGurk D, Thomas JL, et al. Mild traumatic brain injury in US soldiers returning from Iraq. N Engl J Med 2008;358(5):453–463; doi: 10.1056/NEJMoa072972 18234750

[173] Agimi Y, Regasa LE, Stout KC. Incidence of traumatic brain injury in the US Military, 2010–2014. Mil Med 2019;184(5–6):e233–e241; doi: 10.1093/milmed/usy313 30517721

[174] DePalma RG, Cross GM, Beck L, et al. Epidemiology of mTBI (mild traumatic brain injury) due to blast: history, DOD/VA data bases: challenges and opportunities. Proceedings of the NATO RTO-MP-HFM-207 Symposium on a Survey of Blast Injury Across the Full Landscape of Military Science, Halifax, Nova Scotia, Canada, October 3–5, 2011.

[175] Agoston DV. Modeling the long-term consequences of repeated blast-induced mild traumatic brain injuries. J Neurotrauma 2017;34(S1):S44–S52; doi: 10.1089/neu.2017.5317 28937952 PMC5610388

[176] Siedhoff HR, Chen S, Song H, et al. Perspectives on primary blast injury of the brain: translational insights into non-inertial low-intensity blast injury. Front Neurol 2022;12:818169; doi: 10.3389/fneur.2021.818169 35095749 PMC8794583

[177] McGlinchey RE, Milberg WP, Fonda JR, et al. A methodology for assessing deployment trauma and its consequences in OEF/OIF/OND veterans: the TRACTS longitudinal prospective cohort study. Int J Methods Psychiatr Res 2017;26(3):e1556; doi: 10.1002/mpr.1556 28211592 PMC5561532

[178] Walker WC, Hirsch S, Carne W, et al. Chronic Effects of Neurotrauma Consortium (CENC) multicentre study interim analysis: differences between participants with positive versus negative mild TBI histories. Brain Inj 2018;32(9):1079–1089; doi: 10.1080/02699052.2018.1479041 29851515

[179] Dismuke-Greer C, Hirsch S, Carlson K, et al. Health services utilization, health care costs, and diagnoses by mild traumatic brain injury exposure: a chronic effects of neurotrauma consortium study. Arch Phys Med Rehabil 2020;101(10):1720–1730; doi: 10.1016/j.apmr.2020.06.008 32653582

[180] Borinuoluwa R, Ahmed Z. Does blast mild traumatic brain injury have an impact on PTSD severity? A systematic review and meta-analysis. Trauma Care 2023;3(1):9–21; doi: 10.3390/traumacare3010002

[181] Belanger HG, Kretzmer T, Vanderploeg RD, et al. Symptom complaints following combat-related traumatic brain injury: relationship to traumatic brain injury severity and posttraumatic stress disorder. J Int Neuropsychol Soc 2010;16(1):194–199; doi: 10.1017/S1355617709990841 19758488

[182] Elbogen EB, Johnson SC, Wagner HR, et al. Violent behaviour and post-traumatic stress disorder in US Iraq and Afghanistan veterans. Br J Psychiatry 2014;204(5):368–375; doi: 10.1192/bjp.bp.113.134627 24578444 PMC4006087

[183] Rosenfeld JV, Ford NL. Bomb blast, mild traumatic brain injury and psychiatric morbidity: a review. Injury 2010;41(5):437–443; doi: 10.1016/j.injury.2009.11.018 20189170

[184] Vasterling JJ, Verfaellie M, Sullivan KD. Mild traumatic brain injury and posttraumatic stress disorder in returning veterans: perspectives from cognitive neuroscience. Clin Psychol Rev 2009;29(8):674–684; doi: 10.1016/j.cpr.2009.08.004 19744760

[185] Van Wingen GA, Geuze E, Caan MW, et al. Persistent and reversible consequences of combat stress on the mesofrontal circuit and cognition. Proc Natl Acad Sci U S A 2012;109(38):15508–15513; doi: 10.1073/pnas.1206330109 22949649 PMC3458361

[186] Elder GA, Mitsis EM, Ahlers ST, et al. Blast-induced mild traumatic brain injury. Psychiatr Clin North Am 2010;33(4):757–781; doi: 10.1016/j.psc.2010.08.001 21093677

[187] Lew HL, Otis JD, Tun C, et al. Prevalence of chronic pain, posttraumatic stress disorder, and persistent postconcussive symptoms in OIF/OEF veterans: polytrauma clinical triad. J Rehabil Res Dev 2009;46(6):697–702; doi: 10.1682/jrrd.2009.01.0006 20104399

[188] DePalma RG, Hoffman SW. Combat blast related traumatic brain injury (TBI): decade of recognition; promise of progress. Behav Brain Res 2018;340:102–105; doi: 10.1016/j.bbr.2016.08.036 27555540

[189] Mac Donald CL, Johnson AM, Wierzechowski L, et al. Prospectively assessed clinical outcomes in concussive blast vs nonblast traumatic brain injury among evacuated US military personnel. JAMA Neurol 2014;71(8):994–1002; doi: 10.1001/jamaneurol.2014.1114 24934200

[190] Hayes JP, Miller DR, Lafleche G, et al. The nature of white matter abnormalities in blast-related mild traumatic brain injury. Neuroimage Clin 2015;8:148–156; doi: 10.1016/j.nicl.2015.04.001 26106539 PMC4473287

[191] Mac Donald C, Johnson A, Cooper D, et al. Cerebellar white matter abnormalities following primary blast injury in US military personnel. PLoS One 2013;8(2):e55823; doi: 10.1371/journal.pone.0055823 23409052 PMC3567000

[192] Miller DR, Hayes JP, Lafleche G, et al. White matter abnormalities are associated with chronic postconcussion symptoms in blast-related mild traumatic brain injury. Hum Brain Mapp 2016;37(1):220–229; doi: 10.1002/hbm.23022 26497829 PMC4760357

[193] Riedy G, Senseney JS, Liu W, et al. Findings from structural MR imaging in military traumatic brain injury. Radiology 2016;279(1):207–215; doi: 10.1148/radiol.2015150438 26669604

[194] Robinson ME, Lindemer ER, Fonda JR, et al. Close-range blast exposure is associated with altered functional connectivity in veterans independent of concussion symptoms at time of exposure. Hum Brain Mapp 2015;36(3):911–922; doi: 10.1002/hbm.22675 25366378 PMC6869346

[195] Edlow BL, Bodien YG, Baxter T, et al. Long-term effects of repeated blast exposure in United States Special Operations Forces personnel: a pilot study protocol. J Neurotrauma 2022;39(19–20):1391–1407; doi: 10.1089/neu.2022.0030 35620901 PMC9529318

[196] Stone JR, Avants BB, Tustison NJ, et al. Functional and structural neuroimaging correlates of repetitive low-level blast exposure in career breachers. J Neurotrauma 2020;37(23):2468–2481; doi: 10.1089/neu.2020.7141 32928028 PMC7703399

[197] Wang Z, Wilson CM, Mendelev N, et al. Acute and chronic molecular signatures and associated symptoms of blast exposure in military breachers. J Neurotrauma 2020;37(10):1221–1232; doi: 10.1089/neu.2019.6742 31621494 PMC7232647

[198] Hellewell SC, Granger DA, Cernak I. Blast-induced neurotrauma results in spatially distinct gray matter alteration alongside hormonal alteration: a preliminary investigation. Int J Mol Sci 2023;24(7):6797; doi: 10.3390/ijms24076797 37047768 PMC10094760

[199] Clark AL, Merritt VC, Bigler ED, et al. Blast-exposed veterans with mild traumatic brain injury show greater frontal cortical thinning and poorer executive functioning. Front Neurol 2018;9:873; doi: 10.3389/fneur.2018.00873 30473678 PMC6237912

[200] Eierud C, Nathan DE, Bonavia GH, et al. Cortical thinning in military blast compared to non-blast persistent mild traumatic brain injuries. Neuroimage Clin 2019;22:101793; doi: 10.1016/j.nicl.2019.101793 30939340 PMC6446073

[201] Lindemer ER, Salat DH, Leritz EC, et al. Reduced cortical thickness with increased lifetime burden of PTSD in OEF/OIF Veterans and the impact of comorbid TBI. Neuroimage Clin 2013;2:601–611; doi: 10.1016/j.nicl.2013.04.009 24179811 PMC3777819

[202] Martindale SL, Shura RD, Rostami R, et al. Research Letter: blast exposure and brain volume. J Head Trauma Rehabil 2021;36(6):424–428; doi: 10.1097/HTR.0000000000000660 33656482

[203] Tate D, York G, Reid M, et al. Preliminary findings of cortical thickness abnormalities in blast injured service members and their relationship to clinical findings. Brain Imaging Behav 2014;8:102–109; doi: 10.1007/s11682-013-9257-9 24100952 PMC4714342

[204] Vartanian O, Coady L, Blackler K, et al. Neuropsychological, neurocognitive, vestibular, and neuroimaging correlates of exposure to repetitive low-level blast waves: evidence from four nonoverlapping samples of Canadian breachers. Mil Med 2021;186(3–4):e393–e400; doi: 10.1093/milmed/usaa332 33135742

[205] Nathan DE, Bellgowan JF, Oakes TR, et al. Assessing quantitative changes in intrinsic thalamic networks in blast and nonblast mild traumatic brain injury: implications for mechanisms of injury. Brain Connect 2016;6(5):389–402; doi: 10.1089/brain.2015.0403 26956452

[206] LaValle CR, Carr WS, Egnoto MJ, et al. Neurocognitive performance deficits related to immediate and acute blast overpressure exposure. Front Neurol 2019;10:949; doi: 10.3389/fneur.2019.00949 31572285 PMC6754066

[207] Kamimori G, Reilly L, LaValle C, et al. Occupational overpressure exposure of breachers and military personnel. Shock Waves 2017;27(6):837–847; doi: 10.1007/s00193-017-0738-4

[208] Nakashima A, Vartanian O, Rhind SG, et al. Repeated occupational exposure to low-level blast in the Canadian armed forces: effects on hearing, balance, and ataxia. Mil Med 2022;187(1–2):e201–e208; doi: 10.1093/milmed/usaa439 33492379

[209] Tate CM, Wang KK, Eonta S, et al. Serum brain biomarker level, neurocognitive performance, and self-reported symptom changes in soldiers repeatedly exposed to low-level blast: a breacher pilot study. J Neurotrauma 2013;30(19):1620–1630; doi: 10.1089/neu.2012.2683 23687938

[210] Carr W, Taylor M, LoPresti M, et al. Symptomology observed in humans following acute exposure to explosive blast. J Neurotrauma 2015;32:A109.

[211] Kamimori GH, LaValle CR, Eonta SE, et al. Longitudinal investigation of neurotrauma serum biomarkers, behavioral characterization, and brain imaging in soldiers following repeated low-level blast exposure (New Zealand Breacher Study). Mil Med 2018;183(suppl_1):28–33; doi: 10.1093/milmed/usx186 29635591

[212] Elster N, Boutillier J, Magnan P, et al. A critical review of experimental analyses performed on animals, postmortem human subjects, and substitutes to explore primary blast-induced Traumatic Brain injuries. Front Mech Eng 2023;9:1185231; doi: 10.3389/fmech.2023.1185231

[213] Ravula AR, Das T, Gosain A, et al. An update on repeated blast traumatic brain injury. Curr Opin Biomed Eng 2022;24:100409.

