# Supplementary material for: Twenty Years of Blast-Induced Neurotrauma: Current State of Knowledge
Source: Neurotrauma Rep. 2024 Mar 14;5(1):243–53. doi: 10.1089/neur.2024.0001 (PMC10956535; doi:10.1089/neur.2024.0001)

**SUPPLEMENTARY FIG. S1.** Experimentally measured intracranial pressures using various experimental head models. Experimental data is based on Goeller et al. (2012), Hua et al. (2014), Selvan et al. (2013) for head surrogate; Bir (2011), Ganpule et al. (2016), Salzar et al. (2017) for PMHS; Bolander et al. (2011), Chavko et al. (2007), Chavko et al. (2011), Dal Cengio Leonardi et al. (2012), Leonardi et al. (2011), Skotak et al. (2013), Sundaramurthy et al. (2012) for rat. (a) peak incident overpressure ($P_{BOP}$) (b) ratio of peak intracranial pressure to the peak incident overpressure$\left( \frac{P_{ICP}}{P_{BOP}} \right)$(c) ratio of peak intracranial pressure to the peak reflected overpressure $\left( \frac{P_{ICP}}{P_{R}} \right).$ Reflected overpressures were estimated based on Rankine-Hugoniot relations (Kinney and Graham 1985) for PMHS and rat testing device (Sedberry et al. 2018) for rat, where reflected overpressure data was unavailable. (d) Time taken to achieve the peak intracranial pressure in the brain. All the experimental data is based on shock tube experiments. The layered configuration of head parenchyma and the thickness of each layer govern the ICPs in the brain. A simple one-dimensional model of head parenchyma can elucidate the experimental trends (Sutar and Ganpule, BMMB, 2020). The figure is reproduced from Sutar and Ganpule, BMMB, 2020, with permission from the publisher.


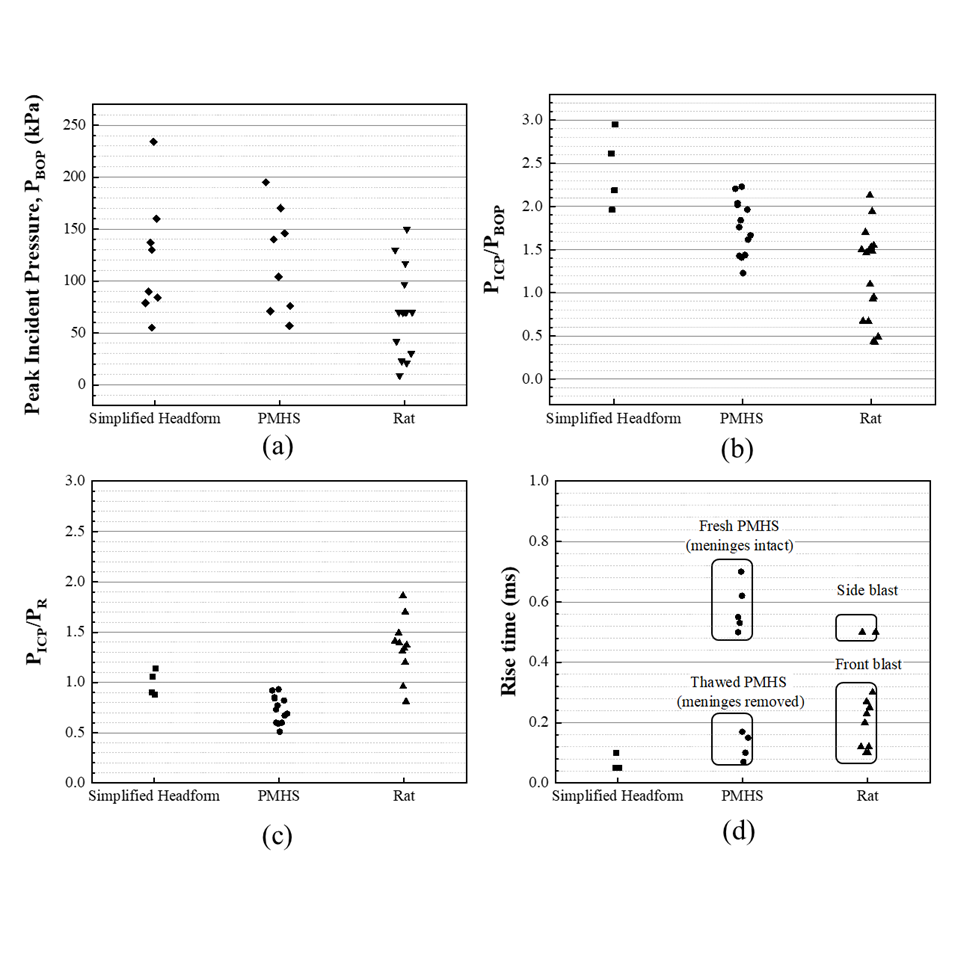

Supplement: Supplemental data [file Suppl_FigS1.docx]
